# Supplementary material for: Testing availability, positioning, promotions, and signage of healthier food options and purchasing behaviour within major UK supermarkets: Evaluation of 6 nonrandomised controlled intervention studies
Source: PLoS Med. 2022 Mar 24;19(3):e1003952. doi: 10.1371/journal.pmed.1003952 (PMC8946731; doi:10.1371/journal.pmed.1003952)
Supplement: S1 Appendix — Table A: Store demographic characteristics (Retailer A). Table B: Store demographic characteristics (Retailer B). Table C: Store demographic characteristics (Retailer C). Table D: Differences in weekly sales of target food categories in intervention versus control stores over the preintervention baseline period. Table E: Average weekly sales of target food categories in intervention versus control stores during the intervention period and comparison of changes before/after intervention between intervention versus control stores. Fig A: ITS analysis showing level and trend changes in weekly sales of products not promoted during the promotional intervention using Disney characters (units/store/week). Table F: Comparison of changes in sales of target food categories (units/store/week) before/after intervention between intervention versus control stores, by store IMD group. IMD, Index of Multiple Deprivation; ITS, interrupted time series. (DOCX) [file pmed.1003952.s002.docx]

# Supplementary Tables and Figures

Contents

[Supplementary Tables and Figures 1](#_Toc89363264)

[Table A. Store demographic characteristics (Retailer A) 2](#_Toc89363265)

[Table B. Store demographic characteristics (Retailer B) 3](#_Toc89363266)

[Table C. Store demographic characteristics (Retailer C) 4](#_Toc89363267)

[Table D. Differences in weekly sales of target food categories in intervention vs control stores over the pre-intervention baseline period 5](#_Toc89363268)

[Table E. Average weekly sales of target food categories in intervention vs control stores during the intervention period and comparison of changes before/after intervention between intervention vs control stores. 6](#_Toc89363269)

[Fig A. Interrupted time series analysis showing level and trend changes in weekly sales of products not promoted during the promotional intervention using Disney characters (Units/store/week) 8](#_Toc89363270)

[Table F. Comparison of changes in sales of target food categories (units/store/week) before/after intervention between intervention vs control stores, by store IMD group. 9](#_Toc89363271)

### Table A. Store demographic characteristics (Retailer A)

| RETAILER A | Total  stores | | Intervention  stores | | Control  stores | | χ^2^ test |
| --- | --- | --- | --- | --- | --- | --- | --- |
| *Frozen chips trial* | **N=180** | **%** | **n=34** | **%** | **n=146** | **%** | **P value** |
| *IMD score groups* |  |  |  |  |  |  |  |
| IMD 1-3 (most deprived) | 51 | 28 | 18 | 53 | 33 | 23 | <0·001 |
| IMD 4-6 | 89 | 49 | 16 | 47 | 73 | 50 |  |
| IMD 7-10 (least deprived) | 40 | 22 | 0 | 0 | 40 | 27 |  |
| *Ethnicity* |  |  |  |  |  |  |  |
| Predominantly white | 44 | 24 | 7 | 21 | 37 | 25 | 0·561 |
| Other ethnicities | 136 | 76 | 27 | 79 | 109 | 75 |  |
| *Fruit & vegetables promotions* | **N=185** | **%** | **N=34** | **%** | **N=151** | **%** | **P value** |
| *IMD score groups* |  |  |  |  |  |  |  |
| IMD 1-3 (most deprived) | 52 | 28 | 18 | 53 | 34 | 23 | <0·001 |
| IMD 4-6 | 91 | 49 | 16 | 47 | 75 | 50 |  |
| IMD 7-10 (least deprived) | 42 | 23 | 0 | 0 | 42 | 28 |  |
| *Ethnicity* |  |  |  |  |  |  |  |
| Predominantly white | 49 | 26 | 7 | 21 | 42 | 28 | 0·388 |
| Other ethnicities | 136 | 74 | 27 | 79 | 109 | 72 |  |

### Table B. Store demographic characteristics (Retailer B)

| RETAILER B | Total  stores | | Intervention  stores | | Control  stores | | χ^2^ test |
| --- | --- | --- | --- | --- | --- | --- | --- |
| *Breakfast cereal positioning* | **N=14** | **%** | **n=7** | **%** | **n=7** | **%** | **P value** |
| *IMD score* |  |  |  |  |  |  |  |
| IMD 1-3 (most deprived) | 5 | 36 | 2 | 29 | 3 | 43 | 0·549 |
| IMD 4-6 | 8 | 57 | 4 | 57 | 4 | 57 |  |
| IMD 7-10 (least deprived) | 1 | 7 | 1 | 14 | 0 | 0 |  |
| *Biscuit range changes* | **N=16** | **%** | **n=8** | **%** | **n=8** | **%** | **P value** |
| *IMD score* |  |  |  |  |  |  |  |
| IMD 1-3 (most deprived) | 7 | 44 | 4 | 50 | 3 | 38 | 0·440 |
| IMD 4-6 | 8 | 50 | 3 | 38 | 5 | 62 |  |
| IMD 7-10 (least deprived) | 1 | 6 | 1 | 12 | 0 | 0 |  |
| *Promotional marketing Disney* | **N=37** | **%** | **n=37** | **%** | **n=0** | **%** | **P value** |
| *IMD score* |  |  |  |  |  |  |  |
| IMD 1-3 (most deprived) | 13 | 35 | 13 | 35 | n/a | n/a | n/a |
| IMD 4-6 | 15 | 41 | 15 | 41 | n/a | n/a |  |
| IMD 7-10 (least deprived) | 9 | 24 | 9 | 24 | n/a | n/a |  |

### Table C. Store demographic characteristics (Retailer C)

| RETAILER C | Total  stores | | Intervention  stores | | Control  stores | | χ^2^ test |
| --- | --- | --- | --- | --- | --- | --- | --- |
| *Shelf labelling beverages* | **N=83** | **%** | **n=18** | **%** | **n=65** | **%** | **P value** |
| *IMD score* |  |  |  |  |  |  |  |
| IMD 1-3 (most deprived) | 21 | 25 | 4 | 22 | 17 | 26 | 0·239 |
| IMD 4-6 | 42 | 51 | 12 | 67 | 30 | 46 |  |
| IMD 7-10 (least deprived) | 20 | 24 | 2 | 11 | 18 | 28 |  |

### Table D. Differences in weekly sales of target food categories in intervention vs control stores over the pre-intervention baseline period

| STRATEGY/TRIAL NAME | Total stores | | Intervention stores | | Control stores | | Student's  t test |
| --- | --- | --- | --- | --- | --- | --- | --- |
| *Availability –*  *Frozen chips trial* | **Mean** | **SD** | **Mean** | **SD** | **Mean** | **SD** | **P value** |
| Regular frozen chips (g) | 14666·6 | 7156·8 | 13035·8 | 6419·3 | 15046·4 | 7285·8 | 0·070 |
| Regular frozen chips (units) | 16·3 | 8·0 | 14·5 | 7·1 | 16·7 | 8·1 | 0·070 |
| Regular frozen chips (£) | 29·0 | 14·3 | 25·8 | 12·8 | 29·7 | 14·5 | 0·074 |
| Total energy from chips (kcal) | 21119·9 | 10305·8 | 18771·5 | 9243·8 | 21666·8 | 10491·6 | 0·070 |
| Total fat from chips (g) | 601·3 | 293·4 | 534·5 | 263·2 | 616·9 | 298·76 | 0·070 |
| *Availability –*  *Biscuit range changes* | **Mean** | **SD** | **Mean** | **SD** | **Mean** | **SD** | **P value** |
| Regular range biscuits (g) | 112381·7 | 24576·5 | 110255·5 | 26420·1 | 114508·0 | 24206·1 | 0·371 |
| Regular range biscuits (units) | 488·9 | 108·1 | 480·2 | 116·5 | 497·6 | 106·3 | 0·380 |
| Regular range biscuits (£) | 577·1 | 135·5 | 575·6 | 136·7 | 578·7 | 143·7 | 0·483 |
| Lower energy range biscuits (g) | 48707·7 | 12166·4 | 50268·0 | 13885·4 | 47147·3 | 10900·4 | 0·688 |
| Lower energy range biscuits (units) | 356·7 | 88·5 | 367·8 | 100·4 | 345·6 | 80·1 | 0·684 |
| Lower energy range biscuits (£) | 449·8 | 120·7 | 471·8 | 133·1 | 427·8 | 111·4 | 0·758 |
| Total energy from biscuits (kcal) | 752245·7 | 168667·8 | 748007·6 | 186598·3 | 756483·8 | 161560·4 | 0·462 |
| *Positioning –*  *Breakfast cereal positioning* | **Mean** | **SD** | **Mean** | **SD** | **Mean** | **SD** | **P value** |
| Regular cereal (g) | 101239·7 | 37166·6 | 109362·5 | 46135·3 | 93117·0 | 26655·6 | 0·782 |
| Regular cereal (units) | 200·8 | 71·3 | 214·7 | 87·4 | 187·0 | 54·1 | 0·755 |
| Regular cereal (£) | 471·0 | 168·3 | 505·6 | 203·8 | 436·4 | 130·4 | 0·768 |
| High fibre cereal (g) | 84292·0 | 30175·8 | 94244·1 | 34396·8 | 74339·9 | 23636·3 | 0·885 |
| High fibre cereal (units) | 168·5 | 76·7 | 195·7 | 99·0 | 141·2 | 34·7 | 0·903 |
| High fibre cereal (£) | 322·8 | 136·3 | 370·5 | 167·1 | 275·0 | 83·7 | 0·899 |
| Total sugars from cereal (g) | 26732·9 | 10168·0 | 29256·7 | 12567·6 | 24209·1 | 7155·7 | 0·813 |
| Total fibre from cereal (g) | 13582·1 | 4933·7 | 14951·4 | 5858·3 | 12212·7 | 3747·6 | 0·841 |
| *Promotions –*  *Promotional marketing Disney* | **Mean** | **SD** | **Mean** | **SD** | **Mean** | **SD** | **P value** |
| Fruits (g) | 27789·8 | 25015·5 | 27789·8 | 25015·5 | n/a | n/a | n/a |
| Fruits (units) | 56·9 | 50·7 | 56·9 | 50·7 | n/a | n/a | n/a |
| Fruits (£) | 62·5 | 56·1 | 62·5 | 56·1 | n/a | n/a | n/a |
| Non sugar baked beans (g) | 9964·5 | 5845·0 | 9964·5 | 5845·0 | n/a | n/a | n/a |
| Non sugar baked beans (units) | 6·0 | 3·5 | 6·0 | 3·5 | n/a | n/a | n/a |
| Non sugar baked beans (£) | 13·0 | 7·5 | 13·0 | 7·5 | n/a | n/a | n/a |
| *Promotions –*  *Fruit & vegetables price promotions* | **Mean** | **SD** | **Mean** | **SD** | **Mean** | **SD** | **P value** |
| Seasonal fruits & vegetables (g) | 267693·9 | 122569·0 | 284445·9 | 95677·7 | 263922·0 | 127814·5 | 0·810 |
| Seasonal fruits & vegetables (units) | 1815·7 | 855·0 | 1926·4 | 690·9 | 1790·8 | 887·9 | 0·798 |
| Seasonal fruits & vegetables (£) | 518·5 | 240·4 | 548·8 | 186·8 | 511·7 | 250·9 | 0·792 |
| *Signage –*  *Shelf labelling beverages* | **Mean** | **SD** | **Mean** | **SD** | **Mean** | **SD** | **P value** |
| Regular beverages (units) | 2095·6 | 771·8 | 1621·3 | 468·5 | 2227·0 | 790·0 | 0·001 |
| Regular beverages (£) | 2040·7 | 781·6 | 1549·6 | 451·5 | 2176·7 | 801·2 | 0·001 |
| Lower/non-sugar beverages (units) | 1800·3 | 732·2 | 1744·3 | 681·2 | 1815·8 | 750·0 | 0·358 |
| Lower/non-sugar beverages (£) | 1565·1 | 589·0 | 1427·0 | 484·1 | 1603·4 | 612·7 | 0·132 |

*Baseline periods: Frozen chip trial 21^st^ Jan – 22^nd^ Sept 2018; Biscuits range 20th May – 12th August 2018; Breakfast cereal 20th May – 12th August 2018; Disney promotions fruits 9th September – 7th October 2018; Disney promotions beans 9th September – 7th October 2018; Fruit & Veg price promotions 29^th^ May – 24^th^ Nov 2018; Shelf labelling beverages 28^th^ May – 27^th^ August 2018.

### Table E. Average weekly sales of target food categories in intervention vs control stores during the intervention period and comparison of changes before/after intervention between intervention vs control stores.

|  | Intervention stores | | | | | Control stores | | | | | *Comparison*  *intervention vs*  *control stores* | | | | |
| --- | --- | --- | --- | --- | --- | --- | --- | --- | --- | --- | --- | --- | --- | --- | --- |
| STRATEGY/TRIAL NAME | **Average sales during**  **intervention period** | | **Absolute difference**  **vs baseline period** | |  | **Average sales during**  **intervention period** | | **Absolute difference**  **vs baseline period** | |  |  |  |  |  |  |
| *Availability –*  *Frozen chips trial* | **Mean** | **SD** | **Mean** | **SD** | **%**  **Change** | **Mean** | **SD** | **Mean** | **SD** | **%**  **Change** | **β**  **/IRR*** | **95%CI** | | **P value** |  |
| Regular frozen chips (g) | 10038·3 | 4726·9 | -2997·5 | 3591·2 | -23% | 14433·8 | 7816·9 | -612·6 | 4061·7 | -4% | -2509·7 | -4059·3 | -960·2 | 0·002 |  |
| Regular frozen chips (units) | 11·2 | 5·3 | -3·3 | 4·0 | -23% | 16·0 | 8·7 | -0·7 | 4·5 | -4% | 0·81 | 0·71 | 0·92 | 0·001 |  |
| Regular frozen chips (£) | 22·2 | 10·5 | -3·5 | 7·1 | -14% | 32·1 | 17·5 | 2·4 | 8·9 | 8% | -5·7 | -9·1 | -2·2 | 0·001 |  |
| Lower fat frozen chips (g) | 3360·9 | 2732·5 | 3360·9 | 2732·5 | n/a | 4·9 | 34·6 | 4·9 | 34·6 | n/a | 3443·0 | 2975·9 | 3910·1 | <0·001 |  |
| Lower fat frozen chips (units) | 3·7 | 3·0 | 3·7 | 3·0 | n/a | 0·0 | 0·0 | 0·0 | 0·0 | n/a | 15356·1 | 2210·8 | 106660·4 | <0·001 |  |
| Lower fat frozen chips (£) | 7·9 | 6·5 | 7·9 | 6·5 | n/a | 0·0 | 0·1 | 0·0 | 0·1 | n/a | 8·1 | 7·0 | 9·3 | <0·001 |  |
| Total energy from chips (kcal) | 19227·7 | 9247·0 | 456·1 | 4548·7 | 2.4% | 2079·.7 | 11249·9 | -875·1 | 5847·792 | -4·0% | 1365·3 | -857·5 | 3588·0 | 0·229 |  |
| Total fat from chips (g) | 505·7 | 238·7 | -28·8 | 125·2 | -5·4% | 591·9 | 320·4 | -24·9 | 166·5 | -4·0% | -4·7 | -67·5 | 58·1 | 0·883 |  |
| *Availability –*  *Biscuit range changes* | **Mean** | **SD** | **Mean** | **SD** | **%**  **Change** | **Mean** | **SD** | **Mean** | **SD** | **%**  **Change** | **β**  **/IRR†** | **95%CI** | | **P value** |  |
| Regular range biscuits (g) | 107030·4 | 24246·6 | -3225·1 | 11321·0 | -3% | 123240·3 | 24164·4 | 8732·3 | 4310·1 | 8% | -13767·9 | -30433·1 | 2897·3 | 0·105 |  |
| Regular range biscuits (units) | 462·6 | 104·2 | -17·6 | 48·3 | -4% | 534·0 | 106·6 | 36·4 | 21·3 | 7% | 0·86 | 0·62 | 1·20 | 0·386 |  |
| Regular range biscuits (£) | 491·1 | 113·3 | -84·4 | 61·5 | -15% | 596·6 | 133·1 | 17·9 | 22·4 | 3% | -104·5 | -184·9 | -24·0 | 0·011 |  |
| Lower energy range biscuits (g) | 58257·4 | 17549·7 | 7989·3 | 7525·4 | 16% | 46953·7 | 13088·5 | -193·6 | 4715·5 | -0·4% | 4339·3 | -1753·5 | 10432·1 | 0·163 |  |
| Lower energy range biscuits (units) | 433·3 | 132·8 | 65·5 | 59·1 | 18% | 339·8 | 96·4 | -5·9 | 33·5 | -2% | 1·11 | 0·93 | 1·32 | 0·245 |  |
| Lower energy range biscuits (£) | 564·0 | 167·5 | 92·2 | 74·6 | 20% | 458·3 | 145·8 | 30·5 | 44·4 | 7% | 24·8 | -35·0 | 84·6 | 0·416 |  |
| Total energy from biscuits (kcal) | 763012·4 | 191952·7 | 15004·7 | 73182·4 | 2% | 796064·5 | 167586·9 | 39580·7 | 32002·7 | 5% | -45850·6 | -148959·2 | 57258·0 | 0·383 |  |
| *Positioning –*  *Breakfast cereal positioning* | **Mean** | **SD** | **Mean** | **SD** | **%**  **Change** | **Mean** | **SD** | **Mean** | **SD** | **%**  **Change** | **β**  **/IRR†** | **95%CI** | | **P value** |  |
| Regular cereal (g) | 110170·1 | 50078·8 | 807·6 | 6847·9 | 1% | 87508·7 | 24079·9 | -5608·3 | 5770·2 | -6% | 98·0 | -5814·4 | 6010·3 | 0·974 |  |
| Regular cereal (units) | 242·4 | 113·7 | 27·7 | 28·3 | 13% | 193·7 | 51·9 | 6·7 | 10·4 | 4% | 1·06 | 0·99 | 1·13 | 0·114 |  |
| Regular cereal (£) | 511·8 | 214·6 | 6·2 | 25·0 | 1% | 412·1 | 124·9 | -24·2 | 22·7 | -6% | 9·66 | -14·10 | 33·42 | 0·425 |  |
| High fibre cereal (g) | 90627·6 | 33183·7 | -3616·5 | 5320·6 | -4% | 75923·7 | 23089·4 | 1583·8 | 8874·3 | 2% | -7646·3 | -16069·4 | 776·7 | 0·075 |  |
| High fibre cereal (units) | 191·2 | 90·0 | -4·6 | 13·1 | -2% | 149·5 | 38·7 | 8·3 | 13·5 | 6% | 1·02 | 0·86 | 1·21 | 0·802 |  |
| High fibre cereal (£) | 398·9 | 166·3 | 28·4 | 18·1 | 8% | 326·1 | 97·8 | 51·1 | 30·1 | 19% | -16·27 | -45·26 | 12·73 | 0·272 |  |
| Total sugars from cereal (g) | 30488·3 | 13344·6 | 1231·6 | 1314·1 | 4% | 24901·5 | 7357·3 | 692·4 | 1336·3 | 3% | -623·2 | -1596·3 | 349·9 | 0·209 |  |
| Total fibre from cereal (g) | 14254·0 | 5759·3 | -697·4 | 819·7 | -5% | 11671·8 | 3425·8 | -540·9 | 967·4 | -4% | -712·7 | -1539·6 | 114·2 | 0·091 |  |
| *Promotions –*  *Promotional marketing Disney* | **Mean** | **SD** | **Mean** | **SD** | **%**  **Change** | **Mean** | **SD** | **Mean** | **SD** | **%**  **Change** | **β**  **/IRR†** | **95%CI** | | **P value** |  |
| Fruits (g) | 92176·0 | 73915·0 | 64386·2 | 53349·2 | 232% | n/a | n/a | n/a | n/a | n/a | n/a | n/a | n/a | n/a |  |
| Fruits (units) | 230·3 | 177·2 | 173·4 | 134·6 | 305% | n/a | n/a | n/a | n/a | n/a | n/a | n/a | n/a | n/a |  |
| Fruits (£) | 146·7 | 112·1 | 84·2 | 68·4 | 135% | n/a | n/a | n/a | n/a | n/a | n/a | n/a | n/a | n/a |  |
| Non sugar baked beans (g) | 15315·4 | 8552·8 | 5350·9 | 6980·0 | 54% | n/a | n/a | n/a | n/a | n/a | n/a | n/a | n/a | n/a |  |
| Non sugar baked beans (units) | 9·2 | 5·2 | 3. 2 | 4·2 | 54% | n/a | n/a | n/a | n/a | n/a | n/a | n/a | n/a | n/a |  |
| Non sugar baked beans (£) | 23·3 | 12·9 | 10·3 | 10·4 | 79% | n/a | n/a | n/a | n/a | n/a | n/a | n/a | n/a | n/a |  |
| *Promotions –*  *Fruit & vegetables price promotions* | **Mean** | **SD** | **Mean** | **SD** | **%**  **Change** | **Mean** | **SD** | **Mean** | **SD** | **%**  **Change** | **β**  **/IRR*** | **95%CI** | | **P value** |  |
| Seasonal fruits & vegetables (g) | 311640·4 | 122470·8 | 27194·5 | 50412·6 | 10% | 271070·4 | 155025·5 | 7148·4 | 51942·9 | 3% | 12019·5 | -6508·3 | 30547·3 | 0·204 |  |
| Seasonal fruits & vegetables (units) | 2118·9 | 799·5 | 192·6 | 283·9 | 10% | 1925·1 | 1004·8 | 134·3 | 283·6 | 8% | 1·1 | 1·0 | 1·2 | 0·101 |  |
| Seasonal fruits & vegetables (£) | 612·0 | 233·8 | 63·2 | 91·1 | 12% | 536·3 | 296·6 | 24·6 | 93·8 | 5% | 22·39 | -10·92 | 55·70 | 0·188 |  |
| *Signage –*  *Shelf labelling beverages* | **Mean** | **SD** | **Mean** | **SD** | **%**  **Change** | **Mean** | **SD** | **Mean** | **SD** | **%**  **Change** | **β**  **/IRR†** | **95%CI** | | **P value** |  |
| Regular beverages (units) | 1604·2 | 443·8 | -17·1 | 223·8 | -1% | 2137·7 | 801·5 | -89·3 | 156·1 | -4% | 0·98 | 0·93 | 1·03 | 0·451 |  |
| Regular beverages (£) | 1605·3 | 413·3 | 55·7 | 220·3 | 4% | 2165·9 | 785·6 | -10·8 | 125·1 | 0% | 38·8 | -28·8 | 106·4 | 0·261 |  |
| Lower/non-sugar beverages (units) | 1406·7 | 467·8 | -337·6 | 286·5 | -19% | 1566·5 | 611·8 | -249·3 | 225·2 | -14% | 0·94 | 0·88 | 1·00 | 0·063 |  |
| Lower/non-sugar beverages (£) | 1406·7 | 467·8 | -20·3 | 174·1 | -1% | 1566·5 | 611·8 | -36·8 | 101·0 | -2% | 24·7 | -22·0 | 71·3 | 0·300 |  |

Baseline periods: Frozen chip trial 21^st^ Jan – 22^nd^ Sept 2018; Biscuits range 20th May – 12th August 2018; Breakfast cereal 20th May – 12th August 2018; Disney promotions fruits 9th September – 7th October 2018; Disney promotions beans 9th September – 7th October 2018; Fruit & Veg price promotions 29^th^ May – 24^th^ Nov 2018; Shelf labelling beverages 28^th^ May – 27^th^ August 2018

* β coefficients from hierarchical normal mixed models (used in the models of gr and £ sales), with fixed effect adjustment for store ethnicity, index of multiple deprivation, affluence and average sales per week over the 2018 period; IRR from hierarchical negative binomial models (used in the models of unit sales), with fixed effect adjustment for store ethnicity, index of multiple deprivation, affluence and average sales per week over the 2018 period.

**†** β coefficients from hierarchical normal mixed models (used in the models of gr and £ sales), with fixed effect adjustment for index of multiple deprivation and average sales per week over the 2018 period; IRR from hierarchical negative binomial models (used in the models of unit sales), with fixed effect adjustment for index of multiple deprivation and average sales per week over the 2018 period.

### Fig A. Interrupted time series analysis showing level and trend changes in weekly sales of products not promoted during the promotional intervention using Disney characters (Units/store/week)

| Regular baked beans 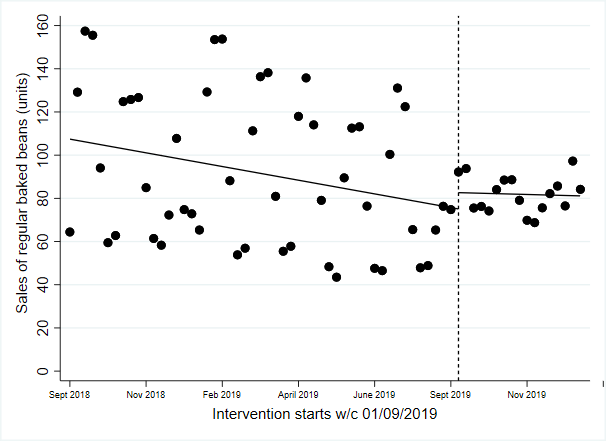 P level=0·323; P trend=0·275 | Other fruits (e.g. normal oranges, bananas) 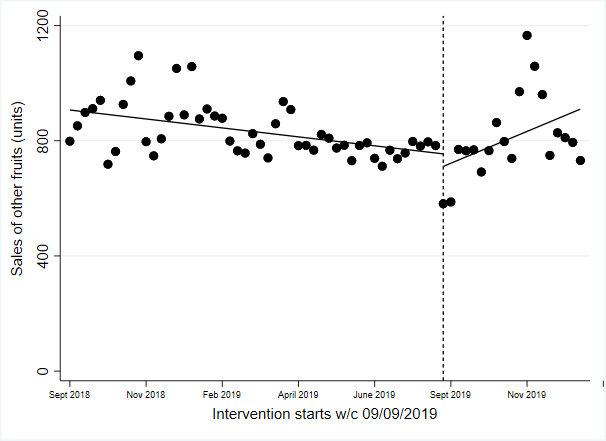 P level=0·460; P trend=0·044 |
| --- | --- |

### Table F. Comparison of changes in sales of target food categories (units/store/week) before/after intervention between intervention vs control stores, by store IMD group.

| STRATEGY/TRIAL NAME | *Intervention vs control stores* | | | | |
| --- | --- | --- | --- | --- | --- |
| *Availability – Frozen chips trial* | **IRR*** | **95%CI** | | **P value** | **P interaction†** |
| Regular frozen chips (units) |  |  |  |  |  |
| IMD 1-3 – higher deprivation | 0·89 | 0·73 | 1·08 | 0·251 | 0·267 |
| IMD 4-10 – middle/lower deprivation | 0·74 | 0·63 | 0·87 | 0·000 |  |
| *Availability – Biscuit range changes* | | | | | |
| Regular range biscuits (units) |  |  |  |  |  |
| IMD 1-3 | 0·86 | 0·58 | 1·27 | 0·445 | 0·839 |
| IMD 4-10 | 0·90 | 0·63 | 1·31 | 0·592 |  |
| Lower energy range biscuits (units) |  |  |  |  |  |
| IMD 1-3 | 1·42 | 1·13 | 1·79 | 0·003 | 0·066 |
| IMD 4-10 | 1·07 | 0·88 | 1·29 | 0·514 |  |
| *Positioning – Breakfast cereal positioning* | | | | | |
| Regular cereal (units) |  |  |  |  |  |
| IMD 1-3 | 1·06 | 1·00 | 1·11 | 0·039 | 0·951 |
| IMD 4-10 | 1·05 | 0·97 | 1·14 | 0·196 |  |
| High fibre cereal (units) |  |  |  |  |  |
| IMD 1-3 | 0·88 | 0·74 | 1·06 | 0·173 | 0·629 |
| IMD 4-10 | 0·97 | 0·88 | 1·07 | 0·530 |  |
| *Promotions – Fruit & vegetables price promotions* | | | | | |
| Seasonal fruits & vegetables (units) |  |  |  |  |  |
| IMD 1-3 | 1·00 | 0·89 | 1·12 | 0·996 | 0·162 |
| IMD 4-10 | 1·12 | 1·00 | 1·26 | 0·048 |  |
| *Signage – Shelf labelling beverages* | | | | | |
| Regular beverages (units) |  |  |  |  |  |
| IMD 1-3 | 0·93 | 0·86 | 1·01 | 0·069 | 0·655 |
| IMD 4-10 | 0·98 | 0·92 | 1·05 | 0·585 |  |
| Lower/non-sugar beverages (units) |  |  |  |  |  |
| IMD 1-3 | 0·97 | 0·84 | 1·11 | 0·621 | 0·577 |
| IMD 4-10 | 0·93 | 0·87 | 1·01 | 0·068 |  |

* IRR from hierarchical negative binomial models (used in the models of unit sales), with fixed effect adjustment for store ethnicity, index of multiple deprivation, affluence and average sales per week over the 2018 period.

**†** P interaction from likelihood ratio tests
