# Supplementary material for: Testing availability, positioning, promotions, and signage of healthier food options and purchasing behaviour within major UK supermarkets: Evaluation of 6 nonrandomised controlled intervention studies
Source: PLoS Med. 2022 Mar 24;19(3):e1003952. doi: 10.1371/journal.pmed.1003952 (PMC8946731; doi:10.1371/journal.pmed.1003952)
Supplement: Attachment — Submitted filename: Response to reviewers CGF 6 trials 02 12 21.docx [file pmed.1003952.s003.docx]

| Requests from the editors: | | |
| --- | --- | --- |
| Comment | Response | Changes |
| Please use the reviewers’ comments to guide your structuring of the revised manuscript. It was generally felt that a more consistent and systematic presentation of the various interventions would facilitate interpretation of your findings. Reviewer 1 suggests that Supplementary tables 1 and 2 could be combined and presented as Table 1 in the main text to facilitate reader comprehension. | We have included as much information as possible about the different interventions and have moved Suppl tables 1 and 2 into the main text | Supplementary tables 1 and 2 were combined into Table 1 in the main manuscript |
| Please update your title such that the setting occurs before the colon ("Natural experiments testing availability, positioning, promotions and signage of healthier food options within major UK supermarkets: Analysis of six non-randomised controlled trials"). | We have changed the title |  |
| Please add a final line to your abstract ‘Methods and findings’ which summarises the main limitations (it should begin ‘The limitations of this study include’ or similar). Given that the manuscript covers a variety of studies, it may be best to focus on the limitations relating to study design more generally as opposed to detailing limitations specific to any one of the experiments presented. | We have added a brief mention to limitations in the abstract | Abstract:  *“These were all retailer-led interventions which present limitations regarding the lack of randomisation, residual confounding from unmeasured variables, absolute differences in trends and sales between intervention vs control stores and no independent measures of intervention fidelity*.*”* |
| Please include line numbering in the margins throughout. | We have added line numbering |  |
| Citations should occur before punctuation. | We have corrected this |  |
| As reflected in the reviewers’ reports, please provide further clarification as to the definition/classification of ethnicity in your study and consider discussing it as a limitation. | We have clarified this in the methods and limitation sections. Unfortunately the information provided by the retailer on consumer ethnicity is considered sensitive and therefore the level of detail they provided to us was limited. | In Methods:  “Ethnicity *of the store customer population was classified by the retailer using internal proprietary systems, and grouped as predominantly white vs other-ethnicities*.”  In Discussion:  *“Finally, there was limited data on store characteristics, and the retailer provided only restricted data on the ethnicity of the customer population. The very broad categorisation of ethnicity is unlikely to have removed all of the confounding related to ethnicity in our results, although there were no significant differences in the distribution of ethnicity between intervention and control stores.”* |
| The PDF conversion process appears to have introduced some minor formatting errors – an example can be found in line 1 of page 7 line 3 (a boxed question mark appears where it should not). Please check throughout for other errors. | We have corrected this |  |
| Please remove the funding information from the acknowledgements section (in the event of publication this will be published as metadata based on your responses to the submission form).  Please also remove the Contributors, Competing interests, and Data sharing sections from the main text for the same reason as above. | We have removed this |  |
| Please remove the Ethical approval statement from the end of the main text as this is already stated in the methods section. | We have removed this |  |
| In your references, please only use et al. after listing the first six authors. Our guidelines can be found here: <https://journals.plos.org/plosmedicine/s/submission-guidelines#loc-references> | We have formatted the references accordingly |  |
| Please provide a completed STROBE checklist. When completing the checklist, please use section names and paragraph numbers rather than page numbers. | We have uploaded a STROBE checklist |  |
| Please reference the checklist in your methods ("This study is reported as per the Strengthening the Reporting of Observational Studies in Epidemiology (STROBE) guideline (S1 Checklist)." | We have added this to the methods section |  |
| When reporting p-values, please use P<0.001 rather than P<0.0001 (please also check your figures and tables to ensure P<0.001 is used consistently). | We have corrected this |  |
| Comments from the reviewers: |  |  |
| Reviewer #1: This is an interesting attempt to analyse six non-randomised controlled trials within major UK supermarkets in one paper on testing availability, positioning, promotions and signage of healthier food options. The study design and statistical methods and analyses are mostly adequate. However, there are a few major issues needing attention. | We thank the reviewer for their positive comments and useful suggestions |  |
| 1) In figure 1, only 1 of 7 trials in stores (intervention vs control) is statistically significant while most trials aren't, which suggests the authors need to tone down all the claims in the paper including in the abstract and discussion. Overall, the trials results are not conclusive with majority of non-significant results therefore the intrepretations need to be balanced, modest and fair. Although the ITS analyses identified a few significant changes before and after intervention, it's mainly technical and secondary and can't substitute the main findings in figure 1. Also it's a bit confusing as why there are 7 trials in figure 1 but throughout the paper only 6 trials are mentioned? | We apologise for the confusion in the presentation of results in Figure 1. This figure includes 8 rows of results corresponding to 5 of the 6 trials analysed in this paper. The 6^th^ trial (Disney promotional activities) did not have control stores because it was rolled out nationwide, therefore can’t be analysed using the hierarchical mixed models.  We disagree though with the reviewer view that the ITS models were secondary. Both methods (hierarchical mixed models and ITS models) were pre-specified a priori in our analytical approach of the primary outcomes. This approach follows our previous publication (Piernas et al, Plos Medicine; <https://doi.org/10.1371/journal.pmed.1003715>) for which the statistical analysis plan was developed by a senior statistician.  Both the hierarchical model and the ITS model are useful to understand the impact of an intervention, but each approach is different. The hierarchical model is used to test average differences in sales between intervention and control stores over the entire period of time while the intervention is implemented (adjusting for baseline sales). The ITS is used to compare differences in sales between intervention vs control stores at the exact time of intervention implementation (referred as level change), and in the trend afterwards.  We have made these differences clearer in the methods. | We have added a footnote to Figure 1 to clarify where the data presented is coming from. |
| 2) Analysing 6 trials in one paper is a bit challenging and the writing sometimes is difficult to follow. Without good and clear understanding of the designs of these 6 store trials, it's difficult to continue to read. However, supplementary table 1 and 2 are extremely informative on design, sample size and intervention of these trials. I'd like to suggest to combine supplementary table 1 and 2 to make it Table 1 in the main paper so that the readers can understand the framework of the trials and then can read the results in the context of design, sample size, intervention and etc. Also, it would be useful to match and align the trials/interventions between figure 1 and new table 1 so that the trial results match the design and sample size. | We have included all this information into a new Table 1 as suggested.  We have added a footnote to Figure 1 to clarify the presentation of the results which matches Table 1 | New Table 1  Footnote in Figure 1 |
| 3) Sample size. From the supplementary table 1, we can see only two trials from retailer A have relatively decent sample size (34 vs 151 and 34 vs 146). All the rest trials have relatively sample size (No. of stores) especially 3 trials from retailer B (8 vs 8 and 7 vs 7 and 37 (no control)). The authors might argue there are many data points (weekly and etc) available but it is really the number of stores that matters. The small sample size may contribute to the mostly non-significant results of these 6 trials as lack of statistical power. This issue needs to be properly discussed as a limitation. | We have included a sentence in limitation highlighting the small samples studied and lack of control stores for some of the trials | In discussion:  “*However, the sample of stores was smaller (<8 stores) for 2 trials and in one case did not include control stores, limiting the power to detect any significant effects*”. |
| 4) The presentation of p-values needs to be consistent throughout the paper. For example, in the abstract, it appear 'P <0·0001' but all the other p-values only have 3 digits. Can authors please check and make sure p-values are presented with 3 digits throughout the paper? | We have changed all p values to 3 decimal places |  |
|  |  |  |
| Reviewer #2: I was asked to review these papers together. Given their similarity many of my comments apply equally to both and so I have provided a combined report. I've noted where comments refer particularly to one or the other paper and described these as the 'combined' or 'easter' papers. Overall, I wasn't convinced by the value of separating these papers. Whilst the combined paper is certainly complicated, I don't think that adding the easter analyses would make it appreciably more so. I think perhaps the only value of the distinct easter paper is that it speaks very specifically to current UK public health policy decisions. On the other hand, the specific high value placement message could be drawn out in post-publication dissemination and, arguably, UK public health policy needs to be thinking beyond just the current policy decisions on the table to some of the strategies presented in the combined paper. | We appreciate the reviewer views about the combination of results into a single paper. This is something we debated at length within the author group. As the reviewer notes, the analyses are incredibly complex and presenting results from multiple interventions makes it more so. We appreciate these trials share similarities, however they also have very important differences.  We decided to separate the intervention on confectionery from the rest because this intervention is the only one within the group that does not involve an implicit swap for a healthier option, this intervention aims to reduce the absolute amount of confectionery sold in stores. In contrast, the rest of the trials aimed to increase sales of healthier food options, through encouraging switches from less healthy to healthier options.  Secondly, the data we received to evaluate each trial was different for the confectionery trial. This is the only study for which we have data on the nutrient content of the overall shopping basket, and so we are able to examine potential compensatory effects. This is a much more robust analysis, hence singling it out. For the other six trials, we could only analyse the total sales of the specific category that was the target of the intervention. The remaining interventions are individually limited by the available data, but as reviewers note, by bringing them together we can comment on the relative impact of different behavioural mechanisms.  The confectionery trial is especially relevant for policy in light of the coming legislation and we believe the messages would get diluted if the manuscripts were combined. | In discussion with the editorial team it was agreed that the manuscripts would stay separate and changes suggested by the reviewer will be made to each where appropriate.  We have extracted the comments from the reviewer which relate specifically to this paper or to both. |
| These papers report on a rather disparate set of interventions in supermarkets aiming to improve the healthiness of purchases. I applaud the authors on the partnership they have developed with the respective retailers and agree that this represents a substantial achievement both in terms of interventions achieved and in data made available. Unfortunately I found the report (particularly in the combined paper) of, what is undoubtedly a complicated collection of work, rather hard to follow and a little underdeveloped in terms of both theoretical introduction and interpretation of findings. | We have included more information regarding the trial design and interventions in table 1 of the current paper (combined paper). We have also revised the Introduction to mention behavioural mechanisms behind these interventions. | In Introduction:  “*Choice architecture interventions in physical micro-environments, such as grocery stores, have been identified and classified in the typology of interventions in proximal physical micro-environments TIPPME framework [12]. Interventions based on placement and availability operate by increasing the range, variety and number, as well as the visibility and accessibility of certain products, while price and promotional strategies can make products cheaper or increase their attractiveness, and systematic review evidence has shown these strategies can help stimulate purchases [13-23].*” |
| The nature of the partnership between the researchers and the retailers is a little unclear. Although there are a number of different definitions of natural experiments, and natural experimental evaluations, key is that the nature and implementation of the intervention is outwith control of the researchers. It is not clear if this was the case here. If there is uncertainty in definitions, non-randomised controlled trials is an unambiguous description of what was done. | The trials were completely organised and implemented by the retailer without any involvement from the study team, therefore completely out of our control. We were invited as an evaluation partner. Hence naming them natural experiments. We have clarified the nature of the partnership. | In Methods:  “*These trials were completely developed and implemented by retail partners, so we followed the methods employed in the monitoring and evaluation of a natural experiment*.” |
| I'd also like to understand more about why this particular group of interventions was selected for study. Was this informed by theory, pragmatic considerations or something else? A key limitation on how the findings can be interpreted is the possibility of an intervention modality by food category interaction - as different food categories were selected for each intervention modality, we can't tell if the effects reported are specific to the food category, the intervention modality, or the interaction of the two. I'd like to see more consideration given to this throughout.  I also thought there was a missed opportunity to raise the issue of industry versus government led public health initiatives in this paper. Again, it wasn't clear to me whether the decision to implement these interventions was driven by retailers, the industry body mentioned, the charity mentioned, or the researchers. Shedding some further light on this may help illuminate how such interventions might be implemented more consistently. | We thank the reviewer for this useful comment. As said above, both the choice of intervention modality and food category was entirely made by the retailers hence potentially not informed by theory. We have added a brief mention to this in the limitation section.  We have also clarified throughout that these were all retailer-led interventions. However it is very likely that the selection of interventions by retailers was influenced by real or perceived government actions or intentions | In Discussion, limitations section:  “*These interventions were selected, developed and implemented by the retailers, without the direct involvement of the research group. It is not possible to know the extent to which this was influenced by behavioural theory, prior commercial insights or awareness of government thinking, though it is probable that all contributed to greater or lesser extent.”* |
| Whilst a lot of information about the interventions and data used is provided in supplementary material (particularly in the combined paper), I would have liked to see this brought into the main text. The scant descriptions of the interventions on p6 of the combined paper left we many unanswered questions that I didn't feel were appropriate to be left to supplementary material. This includes exactly what products were impacted; how eg lower fat and low sugar are defined; whether products are added or moved from elsewhere in store etc. | We have included a new Table 1 which combines the information from Supplementary tables 1 and 2 | See new Table 1 |
| The application of IMD and ethnicity to stores was poorly explained and, I felt, crude. Was this using lower level super output area of location? If so, how likely is it that supermarket customers are drawn only from the surrounding LSOA? At best, I think the limitations of this aspect of the work need further consideration. Perhaps it should be cast in an 'exploratory' light? | Unfortunately we have very limited information with regards to the ethnicity of the surrounding customer population. This is based on the retailer internal systems which classifies a store as having a predominantly White population of customers vs other ethnicities. For the store IMD, we used the postcode of the store to get information of the IMD decile where the store is located. We have added more information in the methods section about this, as well as an explanation in the discussion section on the limitations of the ethnicity classification provided by the retailer. | In Methods:  “*Store characteristics relating to the customer population included the English index of multiple deprivation (IMD) and ethnicity (only Retailer A, Table 1). The store postcode was matched to the IMD income domain, the official measure of relative deprivation in small areas in England [26]. The store sample covered neighbourhoods from deciles 1-10; re-grouped into IMD 1-3 (most deprived), 4-6 (mid) and 7-10 (least deprived). Ethnicity of the store customer population was classified by the retailer using internal proprietary systems, and grouped as predominantly white vs other-ethnicities*.”  In Discussion:  *“Finally, there was limited data on store characteristics, and only one retailer provided restricted data on the ethnicity of the customer population. The very broad categorisation of ethnicity is unlikely to have removed all of the confounding related to ethnicity in our results although there were no significant differences in the distribution of ethnicity between intervention and control stores.”* |
| I'd recommend both manuscripts are reviewed by a statistician. I wasn't clear what the hierarchical vs ITS analyses were contributing and how I should reconcile the information provided by both. More information on what specific questions these two different strategies addressed would be helpful. I found the inconsistent presentation of different information on the results for each intervention in the combined paper additionally confusing. Eg information on nutritional information and differences in pre-intervention sales are sometimes presented, sometimes not. | The analyses reported in both the confectionery Easter paper and the combined paper have followed the same approach reported in our previous paper (Piernas et al, Plos Medicine; https://doi.org/10.1371/journal.pmed.1003715) for which the statistical analysis plan was developed by a senior statistician.  Both the hierarchical model and the ITS model are useful to understand the impact of an intervention, but each approach covers different time periods. The hierarchical model is used to test average differences in sales between intervention and control stores over the entire period of time while the intervention is implemented (adjusting for baseline sales). The ITS is used to compare differences in sales between intervention vs control stores at the exact time of intervention implementation (referred as level change), and in the trend afterwards.  The information presented in both papers on store sales is consistent across all trials, and we have pre-intervention sales for all the studies showed here. However, the confectionery trial is the only one with available data on nutritional information of all sales, hence the reason for separating this trial from the rest. We have revised the manuscripts to make sure the information is consistent and have included more background on the use of both methods. |  |
| There were some apparent inconsistencies in the results that could do with more explanation and discussion. For example, in the ITS, I wasn't clear why there seemed to be substantial increases in the level for control stores at the point of intervention in many cases - even if not statistically significant, what do these reflect? I also wasn't clear if seasonal differences had been taken into account (both eg seasonal events such as Christmas, and seasonal patterns such as temperature changes in summer). This seems particularly important in the easter paper. Finally, the changes in some, but not all, nutrients of interest in line with sales (in the easter paper particularly, but I think in the combined paper too, but reporting of nutrients here was less consistent) could be explored further - there appears to be some displacement of purchases to other foods, but not total? | There are some trials where the control stores showed increased sales of the target products which is not surprising, especially when this links to seasonal patterns of consumption. This is evident for the promoted seasonal fruits and vegetables (Figure 1 Panel C) where both intervention and control stores showed increased sales at the point of intervention, although the increase in intervention stores was greater.  With regards to seasonality, the most important consideration is that, if seasonality appears to be similar between intervention and control stores, then this should not be a major problem in the analyses. The ITS models show weekly sales where it is evident that seasonal fluctuations appear to follow similar patterns in the intervention and control stores over the time period studied. Additionally, the difference in difference approach further accounts for small time-related fluctuations and reduces the effect of absolute differences between the intervention and control stores in the estimations.  The reporting of changes in nutrients from total sales was only possible for the confectionery trial, reported elsewhere. Hence, for this paper combining the 6 trials, the focus is on sales of the target products, and in some cases where appropriate data was available, we looked at changes in nutrients from sales of specific products as secondary outcomes (e.g. total kcal from frozen chips, etc in Supplementary tables) |  |
| I would like to see more consideration in the discussion section (of the combined paper) of why some intervention modalities seemed more promising than others. The authors present a general background of inconsistent previous literature. What do these findings add? Are we any further forward in working out in what contexts different intervention modalities may be more or less effective? How could we make progress on this? | We have revised the introduction and discussion of the current paper to address the reviewer point. | See various changes to second paragraph of Discussion |
| Overall the manuscripts need attention to terminology (and consistency) of this throughout - eg cost vs value; natural experiment vs natural experimental evaluation; interventions being short-lived vs intervention effects being short lived etc; the 'opposite' of the population approach is the high-risk approach, not the individual approach; some assertions are without reference (eg socioceoncomic inequalities exist); 'hypotheses' are referred to in the results, but none were presented in the aims. | We have revised our manuscript to ensure consistency of the terminology used |  |
| In relation to the consideration of Adams et al (2016), please be clearer that the hypothesis concerning 'agency' is proposed, not stated; and that the agentic effort referred to is specifically of individual potential beneficiaries of interventions - interventions don't have agency, they require individual recipients to use their agency and mobilise their resources in order to benefit. | We have revised this sentence | In Introduction:  “*In addition, it is often proposed that environmental interventions are less likely to exacerbate inequalities than individual level interventions because they require less agency from individuals*” |
|  |  |  |
| Reviewer #3: The authors present the results of a series of observational studies on the effectiveness of various nudges to promote healthier grocery purchases. They rely on data from large chain grocery stores across the UK and examine the preliminary success of low touch interventions such as placing healthier options at eye level, coordinating grocery displays with promotional advertising and using signage and labeling to help direct consumers to healthier alternatives. On the one hand, the data are very noisy and the lack of pure randomization makes the (mostly null) results difficult to interpret. I think the authors do a good job of highlighting the limitations of their approach and tried their best to statistically control for the cluster effects, non-randomization and the reality that relying on grocers/retailers to implement these interventions is noisy business. I also worry less about Type 2 errors than Type 1 errors in this situation. Overall, the results are not terribly surprising -- low touch nudges have very small effects on purchasing behavior and the effects are short-lived. I think it's important for policy makers to know this, especially since publication bias often leads to an over-representation of one-off studies showing big effects of these small interventions over relatively short periods of time. Here, we see much more modest effects and they are short-lived. This suggests consumers are willing to try these items but then revert to their preferred brands because the healthier options are less satisfying. I also liked that the authors were realistic in terms of how likely it is that grocers (if given the option) will comply with directives to give premium shelf space to healthy items that don't sell well. I wish the authors would be more specific in their recommendations -- what do they think would work based on their observations? What should policy makers take away from this set of studies? It's an interesting discussion/debate because we see a lot of proposed legislation directed at making (un)healthy options (less)/more available - which doesn't appear to be all that effective in terms of getting consumers to change their behavior. The authors could also do a better job of tying their results in with results from other studies at grocery stores (see Cardario & Chandon, 2019 for a very comprehensive review) or that focus on healthy eating. | We thank the reviewer for their supportive and useful comments.  We agree that the analyses of natural experiments where everything has been planned/implemented by the retailer is not easy, and we relied on the data (sometimes scant and noisy) that they provided to us. However we tried to investigate any biases and confounding with the data we had. For most trials, the use of both control stores and baseline periods of time (e.g. one year before the intervention implementation) can help overcome the lack of randomisation.  With regards to the last point made, we have revised our discussion to emphasize the implications of our results. And we have included the reference suggested. | In Discussion:  *“Our study provide evidence on the effectiveness of interventions (e.g. availability of healthier options next to regular ones) that can help reduce purchases of less healthy options, especially food categories contributing energy, saturated fat and free sugars to the UK diet[2], for which good quality evidence is particularly scarce. However it also highlights the weak impact of other interventions, such as signage and repositioning of items within aisles. While it is hard to draw generic conclusions from these few trials about the effectiveness of interventions targeting different behavioural mechanisms it is clear that, overall, structural interventions targeting automatic behavioural responses were more effective compared to those targeting conscious decisions, which is generally consistent with recent work[36].*  *It is important to note that the interventions here focused on encouraging swaps from a less healthy to a healthier option or increasing overall sales of healthy foods, such as fruits and vegetables. This is an attractive commercial proposition, but the impact of these interventions on the overall energy content of food purchases is likely to be considerably smaller than interventions which specifically seek to reduce impulse of discretionary purchases, such as the removal of foods high in fat, sugar and salt from prominent locations such as end of aisles [37].”* |
