# Supplementary material for: Testing availability, positioning, promotions, and signage of healthier food options and purchasing behaviour within major UK supermarkets: Evaluation of 6 nonrandomised controlled intervention studies
Source: PLoS Med. 2022 Mar 24;19(3):e1003952. doi: 10.1371/journal.pmed.1003952 (PMC8946731; doi:10.1371/journal.pmed.1003952)
Supplement: Attachment — Submitted filename: Response to reviewers CGF 6 trials 20 01 2022.docx [file pmed.1003952.s004.docx]

| Requests from the editors: | | |
| --- | --- | --- |
| Comment | Response | Changes |
| Please revise your title to ‘Testing availability, positioning, promotions and signage of healthier food options within major UK supermarkets: evaluation of six non-randomised intervention studies’. | We have revised the title accordingly | Title:  “*Testing availability, positioning, promotions and signage of healthier food options within major UK supermarkets: evaluation of six non-randomised controlled intervention studies”* |
| Line 20: Please refer to the studies as ‘non-randomised controlled intervention studies’ (including 'controlled' where appropriate) rather than non-randomised controlled trials (please check throughout the manuscript for consistency). | We have applied this change throughout the manuscript |  |
| Lines 24 and 26: Please amend text to “… was associated with decreases in sales”, or similar at line 26. | We have rephrased this sentence accordingly. We have also revised the entire manuscript to ensure consistency. |  |
| Your author summary requires some trimming – please aim for 2-3 bullet points per section (I appreciate that, given the number of interventions, the ‘What did the researchers find’ section may need to be slightly longer than this, so aiming for no more than nine bullet points (preferably single sentences) across the three sections would be OK. | We have revised the author summary to include 2-3 bullet points |  |
| The final line in your Financial Disclosure (‘We thank the consumer goods…’) would be better placed in an Acknowledgements section at the end of the main text (as it does not pertain to funding received). | We have moved this section from the funding disclosure into the Acknowledgements section |  |
| As in the previous draft, it appears the PDF conversion process has introduced some formatting errors (see line 188 for an example). | Apologies, we have corrected this |  |
| Please avoid the term “effects” at line 434. Refer instead to associations. | We have rephrased this sentence | Discussion, conclusion:  “*Some structural interventions within stores, including availability and promotions were associated with significant changes in food purchasing in the intended direction, although the changes observed with promotions on consumer behaviour may diminish with time and are less likely to be sustainable for retailers over longer time periods*.” |
| Please check your references consistently follow our guidance (https://journals.plos.org/plosmedicine/s/submission-guidelines#loc-references) e.g., use of ‘et al’ only after listing the first six authors. | We have checked all references to follow the guidance |  |
| Comments from the reviewers: |  |  |
| Reviewer #1: Thanks authors for their great effort to improve the manuscript. The authors have comprehensively addressed all my comments. I am satisfied with the response and revision. No further issues needing attention. | We thank the reviewer for their time and supportive comments |  |
| Reviewer #2: Thanks for responding to my previous comments. There are two issues that I still think deserve further attention. | We appreciate the reviewer time in reviewing this paper. |  |
| 1. IMD. I am still not clear at what geographical level IMD was calculated. Was is lower level super output area, middle level super output area, ward, local authority? All of these would involve linking postcode to another geography, but it is not clear from your ms which other geography you have used. I suspect you have used lower level super output area. The have an average population of just 1500 people. Hence the IMD of LSOA of location is likely to be a very poor marker of the deprivation of the people who shop in a store - particularly large out-of-town stores which most people drive to, and city center stores with high non-local usage. I still don't see this point discussed in consideration of the equity of effects seen. | The reviewer is right that the IMD used here reflects the relative deprivation at a small local area level (Lower-layer Super Output Areas) across England which covers an average of approximately 1,500 residents or 650 households. We appreciate this is a very crude measure of SES but it was the only appropriate one given the limited data provided to us by the supermarket (store postcode). We have clarified this in Methods and added a limitation regarding this. | Discussion, limitation section:  “*S*i*milarly, the IMD used as a measure of store deprivation may also be a very crude proxy for the SES status of the customer population, particularly when people drive to larger out-of-town supermarkets or for smaller stores located in city centres with a large proportion of non-local customers*” |
| 2. I still don't see discussion of my previous comment "A key limitation on how the findings can be interpreted is the possibility of an intervention modality by food category interaction - as different food categories were selected for each intervention modality, we can't tell if the effects reported are specific to the food category, the intervention modality, or the interaction of the two. I'd like to see more consideration given to this throughout." The conclusions are stated in terms of modality. On occasion there are examples of categories studied in brackets. But it's not made clear that the effects seen may be specific to the categories studied or a modality x category interaction - ie some intervention modalities only work for some food categories. | We appreciate the reviewer suggestion and apologise for having overlooked this comment.  In light of the results obtained, we based our conclusions on intervention modality rather than food categories. This is because for some modalities we had interventions targeting different food categories. For example, frozen chips and biscuits for availability interventions; and seasonal fruits/veg and beans/fruits for promotional interventions. These intervention modalities were the ones that showed significant changes in the intended direction. Expressing our conclusions in terms of intervention modality is also more relevant for policy.  For the other intervention modalities (which showed no significant effects), we only had one food category, therefore we can’t tell if it’s the intervention modality or the food category what influenced the results.  We have added a sentence about this in the limitation section. | Discussion, strengths and limitation section:  “*Finally, for the most promising interventions we studied the effects in two different food categories (e.g. frozen chips and biscuits for availability interventions; seasonal fruits/vegetables and beans/fruits for promotions), and from this we make inferences on the potential effects of these specific intervention modalities. However, for other interventions, we had data on only one food category (e.g. beverages; breakfast cereal) and it remains to be tested whether the effects are also observed across categories. Indeed, one of the research questions facing this field is whether behaviourally similar interventions are equally effective across food categories or whether there are interactions between intervention modality and food category*.” |
| Reviewer #3: Thanks to the authors for a thoughtful revision and set of comments on both papers. I have read the revised manuscripts, the revision notes, and my previous reviews. I remain positive about both papers, but many of my initial concerns remain. First, I don't think the authors can make the causal claims their titles or discussions of the results suggest. Other reviewers have also pointed this out. In my view, these papers are about seeing how various nudges are playing out in the field not about testing interventions. I equate this to non-experimental data that comes in after a drug that did well in clinical trials has been on the market for a while. You cannot overcome a lack of randomization with statistical massaging and/or a pre-post design. You cannot overcome underpowered studies with statistical massaging either. I don't think the word experiment should appear anywhere these papers. There is no natural experiment, as it would require some kind of exogenous shock that resulted in equivalent stores with equivalent populations be essentially randomized to treatment vs. control. I also have reservations about treating these studies as experimental given how little access the researchers had to interventions that were "selected, developed, and implemented" by the retailers -- who I assume are not experimentalists or data scientists. I understand the authors' desire to make a strong policy argument here and it's impressive to have so much data from several retailers trying to implement various nudges simultaneously. However, given the lack of data that's available for controls and robustness checks, I am very hesitant to sign off on a paper that makes causal claims about the viability of certain interventions. I think there are many interesting insights to be gleaned from the data but I think the authors should tone down their language that these results provide any sort of definitive test. I belabor this point because it's easy for keywords that appear in titles and abstracts to become sound bites for journalists and agenda-driven policy makers who never dig a little deeper into the work to realize that the claims cannot be substantiated by the data. | We thank the reviewer for their time and supportive comments.  In response to these comments and the editorial suggestions above, we have removed all instances referring to natural experiments, trials and effectiveness.  We have reworded the title to refer to this study as a non-randomised controlled intervention study |  |
