# Supplementary material for: Testing availability, positioning, promotions, and signage of healthier food options and purchasing behaviour within major UK supermarkets: Evaluation of 6 nonrandomised controlled intervention studies
Source: PLoS Med. 2022 Mar 24;19(3):e1003952. doi: 10.1371/journal.pmed.1003952 (PMC8946731; doi:10.1371/journal.pmed.1003952)
Supplement: Attachment — Submitted filename: Response to reviewers CGF 6 trials 17 02 2022.docx [file pmed.1003952.s005.docx]

| Requests from the editors: | | |
| --- | --- | --- |
| Comment | Response | Changes |
| Please update the manuscript title to ‘Testing availability, positioning, promotions and signage of healthier food options and purchasing behaviour within major UK supermarkets: evaluation of six non-randomised controlled intervention studies’ (my apologies for omitting this suggestion in my previous revision letter). | We have changed the title accordingly | Title |
| In the Data Availability Statement, please provide further details as to how an interested researcher could establish contact with the retailer to request permission to access the data (this could be via an intermediary such as the University Research Office or the Institutional Review Board – please note that it cannot be via a study author). Feel free to contact me directly to discuss ([cdavidson@plos.org](mailto:cdavidson@plos.org)). | We have modified this statement to clarify how to establish contact with the retailer | This research was conducted according to a framework collaboration agreement  between the University of Oxford and the food retailers. Access to the study dataset by  external researchers is not permitted as this is defined as confidential information in the agreement. Access to the study data by external researchers will require the expressed written consent of the retailer. Please contact [hw@theconsumergoodsforum.com](mailto:hw@theconsumergoodsforum.com).  Access to the statistical code used in this analysis will be reviewed and granted upon request by the Nuffield Department of Primary Care PRimDISC committee (primdisc@phc.ox.ac.uk) |
| Line 27: For consistency with the rest of the sentence, please refer to ‘lower energy biscuits’ rather than ‘healthier’. | We changed this |  |
| Line 46 – please remove the protocol registration details from the abstract. | Removed |  |
| Line 54 – please replace the full stop on this line with a comma (‘…may be effective, but most…’). | We changed this |  |
| Line 56 – please begin the sentence ‘As part of a multi-retailer partnership…’ as a new bullet point. | We changed this |  |
| Line 63 – please begin the sentence ‘However, there was no evidence’ as a new bullet point. | We changed this |  |
| Line 75 – in the interest of brevity, please remove this final bullet from the Author Summary. | Removed |  |
| Line 113 – please updated to ‘…by the retailers and aimed at improving…’. | We corrected this |  |
| There are no corresponding asterisks in Figure 1 despite their use in the legend. I would suggest removing the asterisks in the legend rather than adding them to the figure as I do not feel they are necessary for clarity. | We removed asterisks from the legend |  |
| Related to the above, I would suggest updating the Figure 1 legend to read ‘…estimates coming from five of the interventions included in this study, in the following order: Frozen chip range changes (regular frozen chips); Biscuit range changes (regular range biscuits, lower energy range biscuits)…’ etc. I personally find the use of the alphabetical labels in the legend confusing as it suggests a corresponding label in the figure which is not present. | We changed this |  |
| For the relevant panels in Figure 2, please either show the y-axes beginning at zero or show a break in the axes. | We have modified the plots in Figure 2 | Figure 2 |
| For Table F in S2 Appendix, ensure all symbols in your legend are present in the Table. | We corrected this |  |
| Please remove subheadings from the Discussion section. | Removed |  |
| Line 374: ‘provides’. | We corrected this |  |
| Consider separating the strengths and limitations paragraph into two paragraphs. | We have separated this into two paragraphs |  |
| Please check all references for accuracy against our Submission Guidelines (<https://journals.plos.org/plosmedicine/s/submission-guidelines#loc-references>), in particular those for official reports (there are some issues with the authors listed for references 2, 6, and 7, and references 9 and 25 are also not presented consistently). | We have revised all referenced to ensure consistency |  |
| To help us extend the reach of your research, please provide any Twitter handle(s) that would be appropriate to tag, including your own, your coauthors’, your institution, funder, or lab. | [@OxPrimaryCare](http://www.twitter.com/oxprimarycare) |  |
